# ASFV pD345L protein negatively regulates NF-κB signalling by inhibiting IKK kinase activity

**DOI:** 10.1186/s13567-022-01050-z

**Published:** 2022-04-23

**Authors:** Huan Chen, Zhenzhong Wang, Xiaoyu Gao, Jiaxuan Lv, Yongxin Hu, Yong-Sam Jung, Shanyuan Zhu, Xiaodong Wu, Yingjuan Qian, Jianjun Dai

**Affiliations:** 1 MOE Joint International Research Laboratory of Animal Health and Food Safety, College of Veterinary Medicine, Nanjing Agricultural University, Nanjing, Jiangsu China; 2 China Animal Health and Epidemiology Center, Qingdao, China; 3 Jiangsu Agri-Animal Husbandry Vocational College, Veterinary Bio-pharmaceutical, Jiangsu Key Laboratory for High-Tech Research and Development of Veterinary Biopharmaceuticals, Taizhou, Jiangsu China; 4 School of Life Science and Technology, China Pharmaceutical University, Nanjing, China

**Keywords:** African swine fever virus, pD345L, NF-κB, IKK complex, kinase activity

## Abstract

The NF-κB pathway is an essential signalling cascade in the defence against viral infections, including African swine fever virus (ASFV) infection. ASFV encodes more than 151 proteins via its own transcription machinery and possesses a great capacity to evade or subvert antiviral innate immune responses. Although some of these viral proteins have been reported, many remain unknown. Here, we show that pD345L, an ASFV-encoded lambda-like exonuclease, acts as an inhibitor of cGAS/STING-mediated NF-κB signalling by blocking the IkappaB kinase (IKKα/β) activity. Specifically, we showed that overexpression of pD345L suppresses cGAS/STING-induced IFNβ and NF-κB activation, resulting in decreased transcription of IFNβ and several proinflammatory cytokines, including IL-1α, IL-6, IL-8, and TNFα. In addition, we showed that pD345L acts at or downstream of IKK and upstream of p65. Importantly, we found that pD345L associates with the KD and HLH domains of IKKα and the LZ domain of IKKβ and thus interrupts their kinase activity towards the downstream substrate IκBα. Finally, we showed that pD345L-mediated inhibition of NF-κB signalling was independent of its exonuclease activity. Considering these results collectively, we concluded that pD345L blocks IKKα/β kinase activity via protein–protein interactions and thus disrupts cGAS/STING-mediated NF-κB signalling.

## Introduction

African swine fever (ASF) is caused by African swine fever virus (ASFV), a large enveloped double-stranded DNA virus that infects domestic pigs (*Sus scrofa domestica*) and wild boars (*Sus scrofa*) with a morbidity and mortality rate of up to 100%. Since first reported in Kenya in 1921, ASFV genotype I and genotype II have escaped from Africa to Europe, South America, the Caucasus, and the Russian Federation [1]. ASFV genotype I was successfully eradicated in all countries outside Africa except Sardinia. In August 2018, ASFV genotype II was introduced into China and rapidly spread to almost all Chinese provinces as well as more than ten Asian countries [2]. As a result, millions of pigs were culled, and the pig inventory in these countries was significantly decreased. ASFV not only largely threatens the swine industry and heparin supply worldwide but also causes tremendous social and economic impacts [3]. However, there are no effective vaccines or antivirals available yet. Therefore, it is of great importance to better understand ASFV and its host interactions to gain new insight for its control and for vaccine development.

ASFV, the sole member of the family Asfarviridae and the only known DNA arbovirus, shares a common origin with nucleocytoplasmic large DNA viruses (NCLDVs), including poxviruses, iridoviruses, and mimiviruses [4, 5], and completes its replication cycle and assembles newly synthesized virions in the cytoplasmic viral factory. ASFV has a double-stranded DNA genome of 170–190 kbp that contains over 151 open reading frames (ORFs) [6] encoding structural proteins and a variety of proteins involved in gene transcription, replication, nucleotide metabolism, DNA repair and immune regulation [7]. ASFV primarily targets host monocyte-macrophage lineage cells but only replicates with a very low efficiency and often alters its immunogenicity in immortalized cell lines. As innate immune cells, macrophages have a comparably harsh intracellular environment for engulfed microorganisms. Therefore, ASFV employs a delicate mechanism to modulate the intracellular environment to improve viral replication conditions. For example, macrophages produce reactive oxygen species (ROS) in response to viral infection, which not only generates oxidative DNA lesions destroying viral genome integrity [8, 9] but also inhibits viral capsid assembly and maturation [10]. The base excision repair (BER) pathway is the primary repair system activated in response to ROS-induced DNA damage. Interestingly, ASFV encodes all BER pathway enzymes except DNA glycosylase, including AP endonuclease (pE296R), PCNA-like (pE301R); DNA polymerase X-like (pO174L); Lambda-like exonuclease (pD345L); and DNA ligase (pNP419L). It was reported that pE296R is required for ASFV growth in macrophages but not in Vero cells [11] and that pD345L is indispensable for ASFV propagation [12], suggesting an essential role of the DNA repair system in ASFV replication.

In addition, as a large DNA virus, ASFV possesses multiple strategies to evade host innate immune defences usually initiated by recognition of pathogen-associated molecular patterns (PAMPs) by specific pattern recognition receptors (PRRs), such as cyclic GMP-AMP synthase (cGAS), a cytosolic DNA sensor. cGAS recognizes dsDNA and synthesizes the second message cGAMP, which associates with and activates the endoplasmic reticulum (ER)-localized adaptor molecule Stimulator of Interferon Genes (STING). Once activated, STING translocates from the ER to the Golgi, where it phosphorylates the TANK-binding kinase 1(TBK1) and the IkappaB kinase (IKK) to initiate either the interferon regulatory factor 3 (IRF3)- or NF-κB-mediated innate immune responses. The IKK complex contains two catalytic subunits, IKKα and IKKβ, and one regulatory subunit, IKKγ (NEMO). Generally, IKK acts as a key regulator in the NF-κB-mediated antiviral response, and activation of the IKK complex is a common step in activating NF-kB signalling through canonical and noncanonical pathways [13]. Therefore, IKK is an important target for viral immune evasion.

A few ASFV proteins have been identified to regulate host immune responses. For example, A238L, an IκB homologue, is able to either prevent the binding of NF-κB with its response elements in target gene promoters [14] or suppress the transcriptional activity of NF-κB by interacting with p300/CBP [15]. Both I329L, a viral Toll-like receptor (TLR) homologue, and A528R inhibit IRF3 and NF-κB activation [16]. In addition, DP96R was discovered to inhibit the cGAS-STING signalling pathway by targeting TBK1 and IKKβ through an unknown mechanism [17]. Recently, MGF-505-7R has been shown to suppress the cGAS-STING pathway by promoting autophagy-mediated STING degradation and targeting IRF3 and the IKK complex to inhibit IL-1β and type I IFN production [18, 19]. Moreover, viruses with multigene family 360/530 (MGF360/530) deletion show attenuated virulence and enhanced type I IFN production [20, 21]. The chronic/persistent infection caused by live attenuated viruses indicates that additional ASFV proteins modulating the host immune response remain to be identified.

Importantly, we found that pD345L plays a negative role in the cGAS/STING-mediated NF-κB signalling pathway independent of its exonuclease activity but alters IKKα and IKKβ kinase activity through protein–protein interactions. This suggests that pD345L is important for viral replication in host cells through its DNA repair and immune suppression activities.

## Materials and methods

### Plasmids

For mammalian expression, p3XFLAG-CMV-D345L was kindly provided by Dr Hongjun Chen (Shanghai Veterinary Research Institute). pcDNA3.1-HA-cGAS and pcDNA3-2xFlag-STING were generated as previously described [22]. Pig IKKα (EU399820.1), IKKβ (NM_001099935), TAB1 (NM_001244067), TBK1 (NM_001105292), IκBα (NM_001005150), TAK1 (KU504629.1), and p65 (KC316023.1) were amplified from cDNA of PK15 and cloned into pcDNA4-HA. The D345L catalytic mutant (D345L-D108A, E144A and K146A) was constructed using a homologous recombination kit (Vazyme Biotech, China). The luciferase reporter plasmids used in this study were pGL3-Basic-IFN-β-Luc, pGL4.32-NF-κB-Luc and pGluc-IRF3-Luc, which contain the −296 to + 52 fragment of the pig IFN-β promoter [22], five copies of the NF-κB-response element and four copies of the IRF3-response element, respectively.

### Cells, antibodies and reagents

293 T and PK15 cells were grown in Dulbecco’s modified Eagle’s medium (DMEM; Gibco-BRL) supplemented with 8% foetal bovine serum (PAN-Biotech, Dorset, UK) and 1% penicillin and streptomycin (Beyotime Biotechnology, Shanghai, China) at 37 °C in a 5% CO_2_ incubator. WSL cells were maintained in RPMI 1640 medium (Gibco-BRL) supplemented with 10% foetal bovine serum and 1% penicillin and streptomycin.

The primary antibodies used in this study obtained from Cell Signalling (Beverly, USA) included rabbit anti-phospho-p65 (serine 536), rabbit anti-p65, mouse anti-IκBα, rabbit anti-p-IκBα (serine 32), rabbit anti-IKKα, rabbit anti-IKKβ, and rabbit anti-haemagglutinin (HA) antibodies. A mouse anti-Flag antibody was purchased from Sigma–Aldrich (St. Louis, MO, USA), and a rabbit anti-actin antibody was purchased from Proteintech (Wuhan, China). A polyclonal antibody against pD345L was generated in mice by immunization with purified recombinant C-terminal pD345L protein. HRP-conjugated goat anti-mouse IgG (H + L) or anti-rabbit IgG (H + L) were purchased from Millipore. Alexa Fluor 488-conjugated goat anti-mouse IgG and Alexa Fluor 555-conjugated goat anti-rabbit IgG were purchased from Thermo Fisher Scientific (MA, USA). 2′3′-cGAMP and poly(I:C) HMW were purchased from InvivoGen (San Diego, CA, USA). Recombinant TNFα was purchased from Sigma–Aldrich (St. Louis, MO, USA). The protease inhibitor cocktail was purchased from Thermo Fisher Scientific (MA, USA).

### RNA isolation and semiquantitative PCR

Total RNA was extracted from 293 T, WSL or PK15 cells using a Simple P Total RNA Extraction Kit (Bioer Technology, China) according to the manufacturer’s instructions. Samples were subjected to reverse transcription using a HiScript II Q RT kit (Vazyme Biotech). The cDNA was used as a template for semiquantitative RT–PCR to investigate the effect of pD345L on the mRNA levels of pig IL-1α, IL-6, IL-8, IFNβ, and TNFα in cGAMP untreated or treated WSL or PK15 cells or the effect of pD345L, pD345L-CM, and pD345L-C on the mRNA levels of IFNβ in cGAMP untreated or treated WSL cells. In addition, SYBR green-based quantitative real-time PCR (Vazyme Biotech) was performed to investigate the effect of pD345L on the mRNA levels of IFNβ in 293 T cells co-transfected with pcDNA3.1-HA-cGAS and pcDNA3-2xFlag-STING by using a Life Technology instrument. The primers used are listed in Table 1.

**Table 1:** **Primers used for RT–PCR and qRT–PCR**.

Name	Sequence
sus-IL-1α forward	5′-CAAGGACAGTGTGGTGATGG-3′
sus-IL-1α reverse	5′-TCATGTTGCTCTGGAAGCTG-3′
sus-IL-2 forward	5′-CAAACGGTGCACCTACTTCA-3′
sus-IL-2 reverse	5′-CCTGCTTGGGCATGTAAAAT-3′
sus-IL-6 forward	5′-AAGGTGATGCCACCTCAGAC-3′
sus-IL-6 reverse	5′-TCTGCCAGTACCTCCTTGCT-3′
sus-IL-8 forward	5′-TGGCAGTTTTCCTGCTTTCT-3′
sus-IL-8 reverse	5′- CAGTGGGGTCCACTCTCAAT-3′
sus-TNFα forward	5′- CCACCAACGTTTTCCTCACT-3′
sus-TNFα reverse	5′- CCCAGGTAGATGGGTTCGTA-3′
sus-IFNβ forward	5′- TTGGCATGTCAGAAGCTCCT-3′
sus-IFNβ reverse	5′- CTGGAATTGTGGTGGTTGCA-3′
sus-Actin forward	5′- GAGACCTTCAACACCCCAGCCATG-3′
sus-Actin reverse	5′- GCGACGTAGCACAGCTTCTCCTTG-3′
D345L forward	5′-ATCTCTATGGGCATCTACGTCG-3′
D345L reverse	5′-AAGACCTTCCATCCAAAGTAGC-3′
h-IFNβ forward	5′-TTCACCAGGGGAAAACTCAT-3′
h-IFNβ reverse	5′-TCCTTGGCCTTCAGGTAATG-3′
h-GAPDH forward	5′-CCACCCAGAAGACTGTGGAT-3′
h-GAPDH reverse	5′-TTCAGCTCAGGGATGACCTT-3′

### Transfection and dual-luciferase assay

The dual-luciferase assay was performed in triplicate according to the manufacturer’s instructions. 293 T cells were seeded on 24-well plates (Thermo Scientific) at a density of 1 × 10^5^ cells per well overnight before transfection. Cells were then co-transfected with p3XFLAG-CMV-D345L (200 ng), pGL3- Basic-IFN-β-Luc (200 ng) and the internal control Renilla luciferase vector pCMV-RL (2 ng) for 24 h using Lipofectamine 2000 reagent (Thermo Fisher Scientific, MA, USA). Luciferase activity was measured with a dual-luciferase assay kit (Promega, Madison, WI, USA) and an MD SpectraMax iD5 instrument.

### Coimmunoprecipitation and immunoblotting

293 T cells were co-transfected with p3XFLAG-CMV-D345L (1.5 μg) and pcDNA4-HA-IKKα/β (1.5 μg) for 36 h. Then, the cells were washed with ice-cold PBS and lysed with lysis buffer (50 mM Tris (pH 7.5), 150 mM NaCl, 1 mM EDTA, and 1% NP-40 supplemented with protease inhibitor) and incubated with the anti-Flag or anti-HA antibody together with protein A/G magnetic beads for 2 h at 4 °C. After eight washes with ice-cold lysis buffer, proteins were eluted with SDS sample buffer and analysed by immunoblotting. For immunoblotting, the coimmunoprecipitation sample and 2% whole-cell lysates were analysed by SDS–PAGE and transferred to a polyvinylidene difluoride (PVDF) membrane (Pall Corp.). The membrane was blocked in 3% skim milk in phosphate-buffered saline with Tween 20 (PBST) for 1 h and then incubated with primary antibodies at 4 °C overnight and anti-mouse or anti-rabbit IgG antibody conjugated to HRP.

### Kinase assay

293 T cells were transfected individually with pcDNA3-2XFlag-IKKα, pcDNA3-2XFlag-IKKβ, pcDNA3XFlag-CMV-D345L, or pcDNA4-HA-IκBα for 36 h and then collected for immunoprecipitation assay with the anti-flag or anti-HA antibody. Each immunoprecipitated (purified) protein was dissolved in kinase reaction buffer (25 mM Tris–HCl (pH 7.5), 0.01% Triton X-100, 10 mM MgCl_2_, 0.5 mM Na_3_VO_4_, 2.5 mM DTT, 0.5 mM EGTA, and 50 μM ATP) with or without ATP. Then, equal amounts of the substrate IκBα with or without IKKα/β and pD345L were subjected to the kinase assay in the reaction buffer and incubated at 30 °C for 1 h. An equal amount of 2 × SDS sample buffer was added to the reaction buffer and used for immunoblot analysis.

### Immunofluorescence assay

WSL cells were grown on coverslips and transfected with p3XFLAG-CMV-D345L or empty vector for 36 h followed by treatment with TNFα (40 ng/mL) for 30 min. The cells were fixed and permeabilized with 4% formaldehyde and 0.1% Triton X-100 at 37 °C for 30 min. After washing with glycine-PBS, the slides were blocked with 3% BSA in PBS for 1 h at room temperature. The slides were incubated first with a primary antibody (1:200) for 1 h and then with a secondary antibody (1:500) for 30 min. Nuclei were stained with DAPI, and images were acquired with a Nikon fluorescence microscope (TS100-F; DSRi2).

### ASFV infection

The ASFV China/2018/AnhuiXCGQ strain (GenBank: MK128995.1) was propagated in alveolar macrophages (PAMs) (23). Briefly, 1 × 10^7^ PAM cells were seeded into a 6 cm dish and incubated for 18 h. Cells were infected with ASFV (MOI 1) in serum-free RPMI 1640 medium at 37 °C for 2 h, washed with PBS and maintained in 10% FBS RPMI 1640 medium. After 24 h of infection, the cells were collected for coimmunoprecipitation assays.

### Statistical analysis

All experiments were performed at least three times unless otherwise indicated. Data are presented as the means ± standard deviations (SDs). Statistical significance between groups was determined using Student’s *t* test in GraphPad Prism 7.0 software (La Jolla, CA, USA). *P < 0.05, **P < 0.01, ***P < 0.001.

## Results

### ASFV pD345L attenuates IFNβ production through cGAS/STING-mediated NF-κB signalling

**Figure 1:**
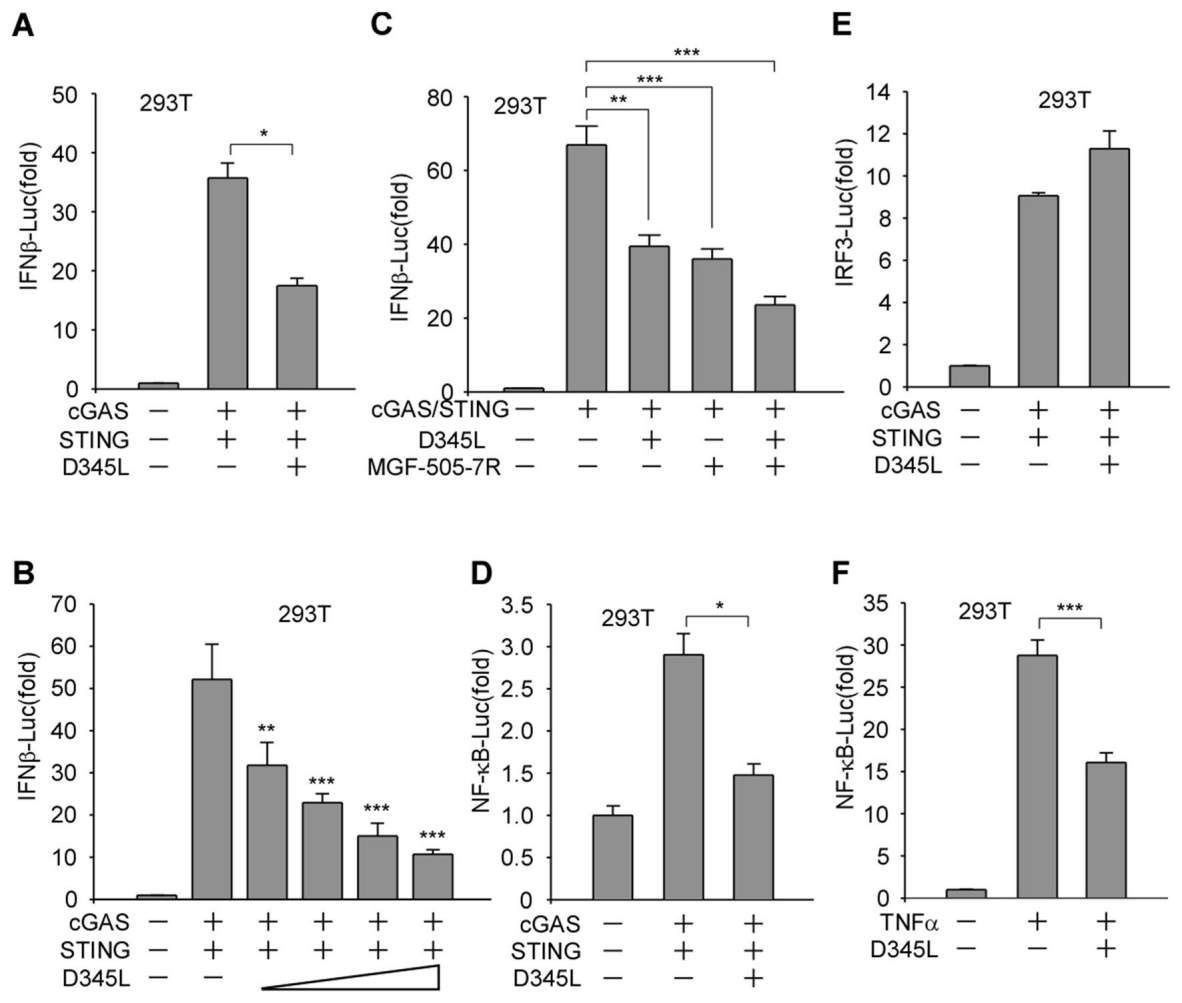
**pD345L inhibits IFNβ expression through cGAS-STING-mediated NF-κB signalling.** **A** 293 T cells transfected with pGL3-Basic-IFNβ-Luc and pCMV-RL along with empty vector, pcDNA3.1-HA-cGAS, pcDNA3-2xFlag-STING and/or p3XFLAG-CMV-D345L for 24 h were collected to measure luciferase activities. **B** The experiment was performed as shown in **A** except using an increasing amount of p3XFLAG-CMV-D345L. **C** 293 T cells transfected with pGL3-Basic-IFNβ-Luc and pCMV-RL along with empty vector, pcDNA3.1-HA-cGAS, pcDNA3-2xFlag-STING and/or p3XFLAG-CMV-D345L, p3XFLAG-CMV-MGF-505-7R for 24 h were collected for measuring luciferase activities. **D** The experiment was performed as shown in **A** except using NF-κB-Luc. **E** The experiment was performed as shown in **A** except using IRF3-Luc. **F** The experiment was performed with 293 T cells transfected with pGL3- Basic-IFNβ-Luc, pCMV-RL and p3XFLAG-CMV-D345L or empty vector for 24 h followed by treatment with TNFα (40 ng/mL) for 6 h. The “-” symbols represent empty vectors for the corresponding coding plasmids.

Upon ASFV infection, cytosolic DNA is mainly detected by the key DNA sensor cGAS [23], activating the STING-dependent type I interferon response. To identify ASFV proteins that regulate the cGAS/STING-mediated immune response, a number of ASFV-encoded proteins were screened using a dual-luciferase reporter assay by transfection of each viral protein with the INFβ, NF-κB or IRF3 luciferase reporter (designated INFβ-Luc, NF-κB-Luc, and IRF3-Luc, respectively), along with co-transfection or treatment with innate immune stimulators. From the results, pD345L was identified. To confirm this, a dual-luciferase reporter assay was conducted with co-transfection of IFNβ-Luc along with cGAS, STING and/or D345L expression vectors, and the results showed that pD345L obviously decreased cGAS/STING-induced activation of the IFNβ promoter in a dose-dependent manner (Figures 1A and B), which was different from the recently published MGF-505-7R protein that inhibits the cGAS/STING pathway (Figure 1C). Next, to further determine whether the NF-κB or IRF3 pathway is targeted by pD345L, the effect of pD345L on NF-κB and IRF3 promoter activation was analysed. The results showed that pD345L suppressed cGAS/STING-induced NF-κB-Luc activity (Figure 1D) but not IRF3-Luc activity (Figure 1E). Similarly, pD345L also inhibited NF-κB-Luc activity induced by TNFα, which activates IKK/NF-κB signalling (Figure 1F). These findings suggest that pD345L inhibits the cGAS/STING-mediated NF-κB signalling pathway.

### pD345L decreases the production of IFNβ and proinflammatory cytokines

**Figure 2:**
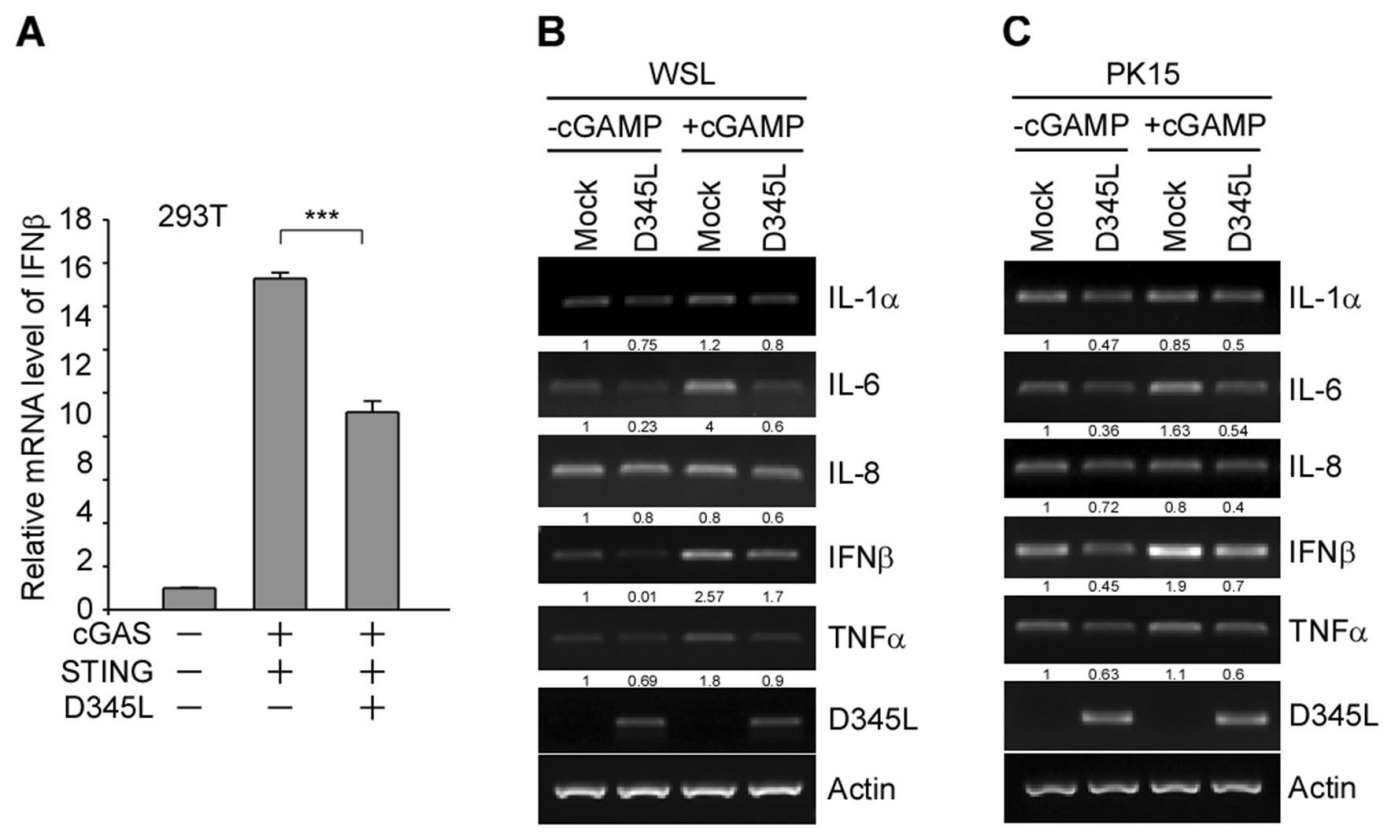
**pD345L suppresses the mRNA expression of IFNβ and proinflammatory cytokines.** **A** Quantitative real-time PCR was performed with RNA extracted from 293 T cells transfected with pcDNA3.1-HA-cGAS and pcDNA3-2xFlag-STING along with p3XFLAG-CMV-D345L or empty vector for 24 h. **B** WSL or **C** PK15 cells were transfected with p3XFLAG-CMV-D345L or empty vector for 30 h followed by treatment with 2′3′-cGAMP (5 μg/mL) for 4 h and then harvested for RNA extraction. Semiquantitative RT–PCR assays were carried out to detect IL-1α, IL-6, IL-8, IFNβ, TNFα, D345L and Actin mRNA expression levels. The levels of IFNβ and proinflammatory cytokines were normalized to the level of actin.

To confirm whether pD345L inhibits IFNβ gene transcription, real-time PCR was performed to examine the effect of pD345L on IFNβ mRNA expression levels upon co-transfection of cGAS/STING with or without D345L in 293 T cells. The results showed that upregulation of IFNβ mRNA induced by cGAS/STING was significantly reduced in the presence of pD345L (Figure 2A). As a key transcription factor controlling the proinflammatory signalling pathway [24], NF-κB controls the expression of a set of proinflammatory cytokines, including I-L1α, IL-6, IL-8 and TNFα. Therefore, if pD345L regulates NF-κB activity, it should also modulate the transcription of NF-κB downstream targets in addition to IFNβ. To test this hypothesis, the mRNA levels of IFNβ as well as four proinflammatory cytokines were examined in WSL cells, an immortalized wild boar lung fibroblast line, and PK15 cells, a porcine kidney cell line, that were left untreated or treated with 2′3’-cGAMP. The results showed that pD345L decreased the mRNA levels of not only IFNβ in both cell lines with or without 2′3’-cGAMP stimulation but also of IL-1α, IL-6, IL-8 and TNFα (Figures 2B and C). Collectively, these results indicate that pD345L inhibits cGAS/STING-mediated NF-κB signalling.

### pD345L suppresses NF-κB activation at or downstream of IKK and upstream of p65

**Figure 3:**
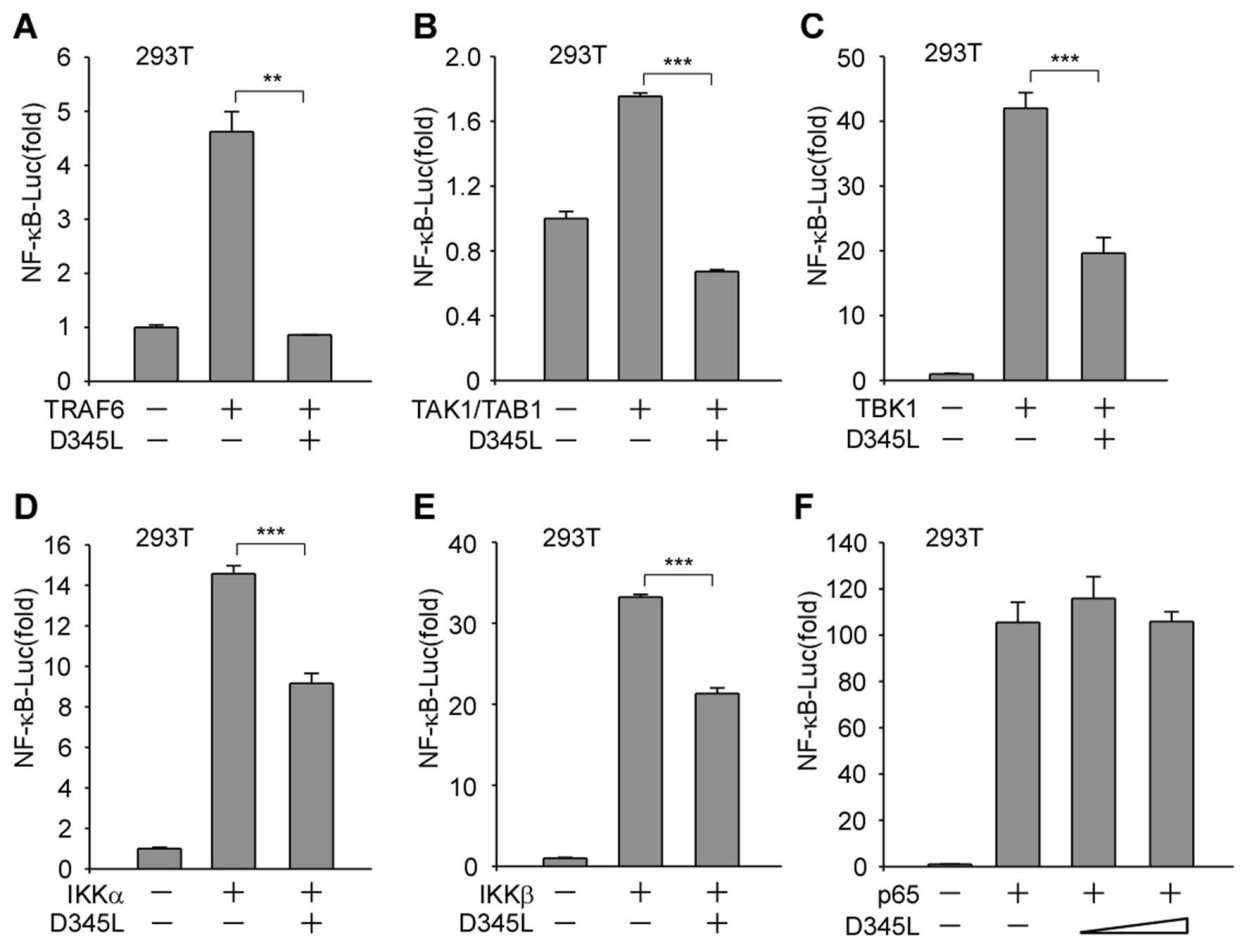
**pD345L suppresses NF-κB activation at or downstream of IKKα/β and upstream of p65.** 293 T cells were transfected with NF-κB-Luc (200 ng), pCMV-RL (2 ng), pcDNA3-Flag-TRAF6 (200 ng) (**A**), pcDNA4-HA-TAK1/TAB1 (each 200 ng) (**B**), pcDNA4-HA-TBK1 (**C**), pcDNA4-HA-IKKα (200 ng) (**D**), pcDNA4-HA-IKKβ (200 ng) (**E**), pcDNA4-HA-p65 (100 ng) (**F**), p3XFLAG-CMV-D345L (200 ng) or empty vector (corresponding quantity). The cells were collected 24 h post-transfection, and luciferase activity was measured.

Upon association of a ligand with its cell surface receptor or recognition of cytosolic DNA by cGAS/STING, adaptors, such as TRAFs, TBK1 or TAK1, will be recruited to the IKK complex to mediate IκBα phosphorylation and degradation and consequently enable translocation of the active NF-κB transcription factor subunits to the nucleus and initiate the expression of its target genes [25, 26]. To characterize the specific target of pD345L, several factors, including TRAF6, TAK1/TAB1, TBK1, IKKα and IKKβ that activate NF-κB at different steps of the signalling cascade were tested. The dual-luciferase reporter assay showed that pD345L was able to abolish NF-κB-Luc activity induced by TRAF6 (Figure 3A) and TAK1/TAB1 (Figure 3B) or significantly suppress NF-κB-Luc activity induced by TBK1 (Figure 3C), IKKα (Figure 3D) or IKKβ (Figure 3E). However, pD345L did not inhibit NF-κB-Luc activity induced by p65 overexpression (Figure 3F). Therefore, it is likely that pD345L modulates NF-κB signalling at or downstream of the IKK complex but upstream of p65.

### pD345L interacts with IKKα and IKKβ

**Figure 4:**
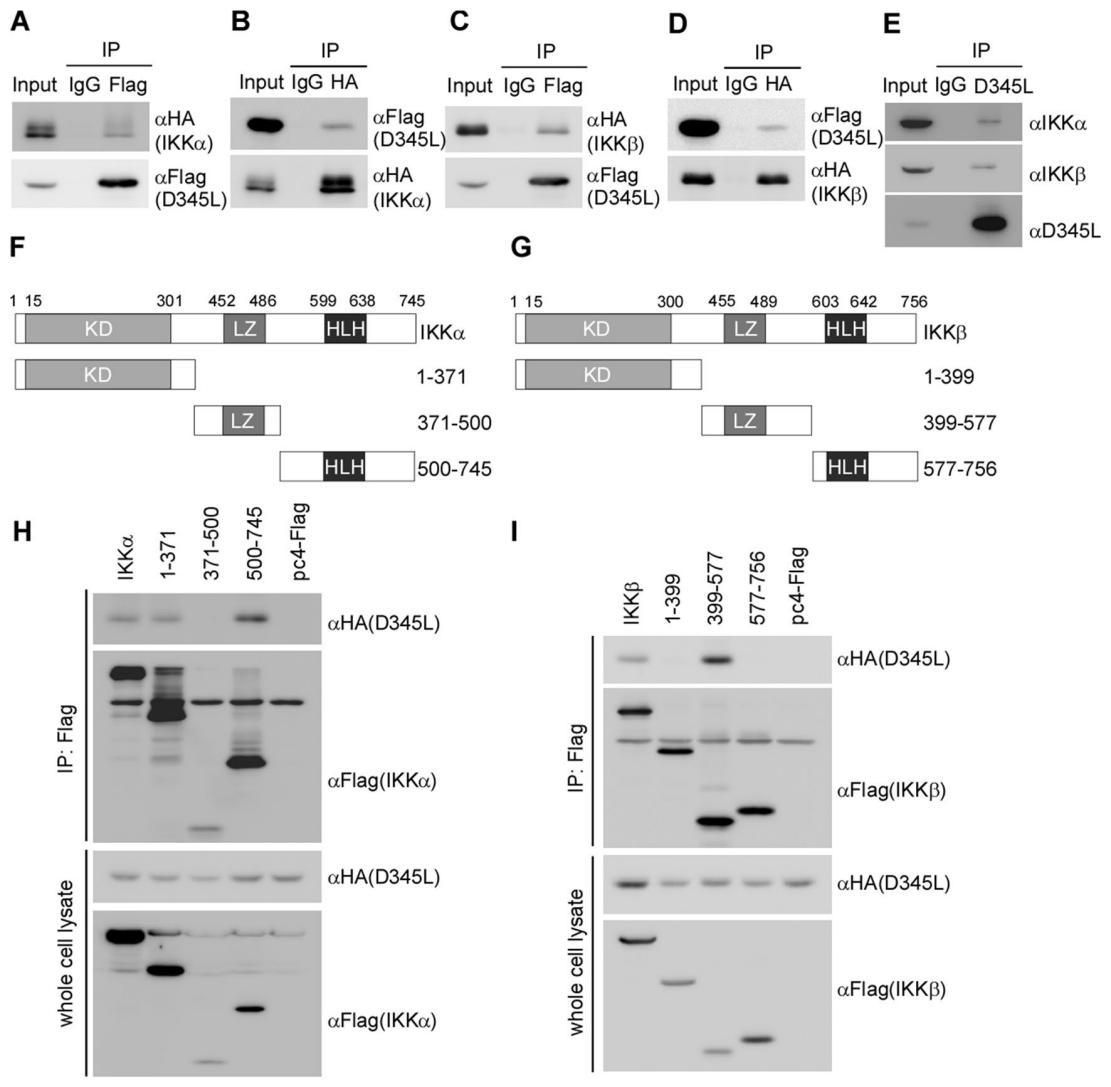
**pD345L interacts with IKKα and IKKβ.** A coimmunoprecipitation assay was performed with whole cell lysates prepared with 293 T cells co-transfected with Flag-D345L and HA-IKKα for 36 h with Flag antibody (**A**), HA antibody (**B**) or control IgG. The immunocomplexes were analysed by immunoblotting with the indicated antibodies. **C** and **D** The experiment was performed as for Panel A or B except using HA-IKKβ. **E** A coimmunoprecipitation assay was performed with whole-cell lysates prepared with PAM cells infected with ASFV (MOI 1) for 24 h with pD345L antibody. The immunocomplexes were analysed by immunoblotting with the indicated antibodies. **F** and **G** Diagrams of truncated IKKα and IKKβ. Both IKKα and IKKβ contain an N-terminal kinase domain (KD), a C-terminal helix–loop–helix domain (HLH) and a leucine zipper domain (LZ), as indicated. The experiment was performed as for **A**, except that HA-D345L and Flag-tagged IKKα, IKKα (1-371), IKKα (371-500), IKKα (500–745), empty vector (**H**), or Flag-tagged IKKβ, IKKβ (1–399), IKKβ (399–577), IKKβ (577–756), empty vector (**I**) were transfected as indicated.

Activation of NF-κB signalling involves the phosphorylation of IκBα by the IKK complex, which includes two catalytic subunits, IKKα and IKKβ, and one regulatory subunit IKKγ [27]. According to the above results, we further explored whether pD345L directly targets IKK to inhibit IκBα phosphorylation. A coimmunoprecipitation assay was performed to analyse the protein–protein interaction between pD345L and IKK by co-transfection of Flag-tagged D345L and HA-tagged IKKα or IKKβ in 293 T cells. The results showed the presence of HA-IKKα (Figure 4A) or HA-IKKβ (Figure 4C) in the anti-Flag antibody-immunoprecipitated pD345L protein complex, and conversely, the presence of Flag-pD345L in the HA antibody-immunoprecipitated IKKα (Figure 4B) or IKKβ (Figure 4D) protein complex. Next, a coimmunoprecipitation assay was performed to further confirm the interactions between pD345L and IKKα/β in ASFV-infected PAM cells. The results showed that endogenous IKKα/β was detected in the pD345L-immunoprecipitated complex (Figure 4E). The IKKα and IKKβ subunits share 52% amino acid sequence identity and have similar primary structures that contain an N-terminal kinase domain (KD), a leucine zipper (LZ) domain and a C-terminal helix-loop-helix (HLH) domain (Figures 4F and G). To determine which domain of IKKα or IKKβ interacts with pD345L, several Flag-tagged truncation mutants containing the KD, LZ or HLH domain of each subunit were constructed as previously described [28] (Figure 4F and G). A coimmunoprecipitation assay was performed and showed that HA-tagged pD345L was detected in anti-Flag antibody-precipitated KD or HLH domain of IKKα (Figure 4H) and LZ domain of IKKβ (Figure 4I), which suggested that pD345L might target IKKα and IKKβ to suppress NF-κB signalling. While the expression level of the IKKα (371–500) truncation mutant was quite low, we cannot rule out the possibility that the IKKα (371–500) truncation mutant could also interact with pD345L.

### pD345L recruits IKKα and IKKβ to suppress their kinase activity towards IκBα

**Figure 5:**
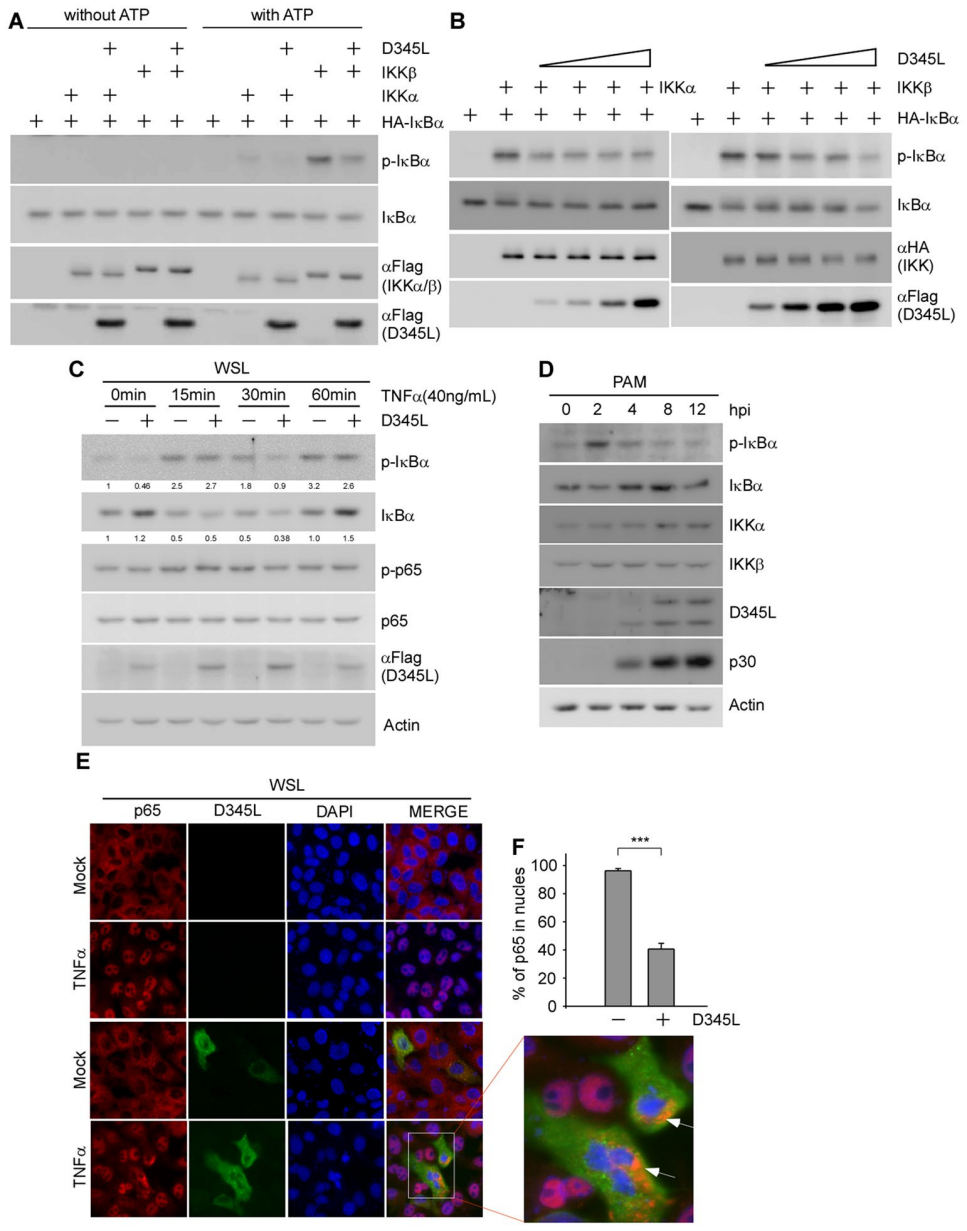
**pD345L targets IKKα/β to inhibit their kinase activity towards IκBα.** **A** 293 T cells were transfected with HA-IκBα, Flag-IKKα, Flag-IKKβ or Flag-D345L. At 36 h post-transfection, these proteins were immunoprecipitated with the anti-Flag or anti-HA antibody. A kinase assay of the immunoprecipitated proteins in vitro was carried out with or without ATP and analysed by immunoblotting. **B** The experiment was performed as for **A** except using an increasing concentration of pD345L in the reaction. **C** WSL cells were transfected with Flag-D345L or empty vector for 30 h followed by treatment with TNFα (40 ng/mL) at the indicated time point. Cells were collected for immunoblotting with the indicated antibodies. **D** PAM cells were mock infected or infected with ASFV (MOI 2) for 2, 4, 8, or 12 h. Whole-cell extracts were prepared and analysed by immunoblotting with the indicated antibodies. **E** WSL cells were transfected with Flag-D345L or empty vector for 36 h followed by treatment with TNFα (40 ng/mL) for 30 min and stained with anti-Flag (green) or anti-p65 (red) antibody. Nuclei were stained with DAPI (blue), and images were acquired with a Nikon fluorescence microscope. **F** Graph shows the intranuclear p65 percentage in pD345L-positive and pD345L-negative cells (TNFα treatment). Data were quantified by counting more than 50 cells in triplicate. The levels of p-IκBα and IκBα were normalized to the level of actin.

KD, LZ and HLH defective mutants of IKKα and IKKβ retain little or no IκBα phosphorylation activity, and either IKKα or IKKβ alone is able to mediate IκBα phosphorylation [29]. Since pD345L interacts with the KD and HLH domains of IKKα and the LZ domain of IKKβ, we suspected that pD345L may directly target IKK to suppress its kinase activity towards IκBα. To address this possibility, the kinase assay was performed with purified pD345L, IκBα, IKKα and IKKβ obtained by immunoprecipitation with or without ATP that provides a phosphate group necessary for the phosphorylation reaction to determine the newly formed p-IκBα. The results showed that in the absence of ATP, almost no p-IκBα was detected, while in the presence of ATP, p-IκBα was obviously increased when reacting with IKKα or IKKβ, but the increase in p-IκBα was prohibited by pD345L (Figure 5A). In addition, upon treatment with an increasing concentration of pD345L, IKKα− or IKKβ-activated p-IκBα was gradually decreased (Figure 5B). To further confirm these results, a D345L expression vector or empty vector was transfected into WSL cells followed by TNFα stimulation at the indicated time points. Immunoblotting results showed that p-IκBα was decreased at 0 and 30 min post-TNFα treatment, and conversely, the total IκBα level was increased at 0 and 60 min post-TNFα treatment (Figure 5C). This suggests that pD345L overexpression inhibited TNFα-induced IκBα phosphorylation, and thus, the total IκBα level was recovered, which would prevent NF-κB release from IκBα. Consistent with these findings, ASFV infection-stimulated NF-κB activation (IκBα phosphorylation) was attenuated along with pD345L expression in PAM cells (Figure 5D). Next, p65 translocation was examined in WSL cells with or without pD345L expression. The results showed that p65 translocated from the cytoplasm to the nucleus after TNFα treatment; however, it remained in the cytoplasm of cells expressing pD345L (Figure 5E). The percentage of intranuclear p65 in pD345L-positive cells was significantly decreased compared with that in pD345L-negative cells (Figure 5F). Therefore, these findings indicate that pD345L directly inhibits IκBα phosphorylation through association with IKKα and IKKβ, further suppressing p65 nuclear translocation.

### pD345L inhibits NF-κB signalling independent of its exonuclease activity

**Figure 6:**
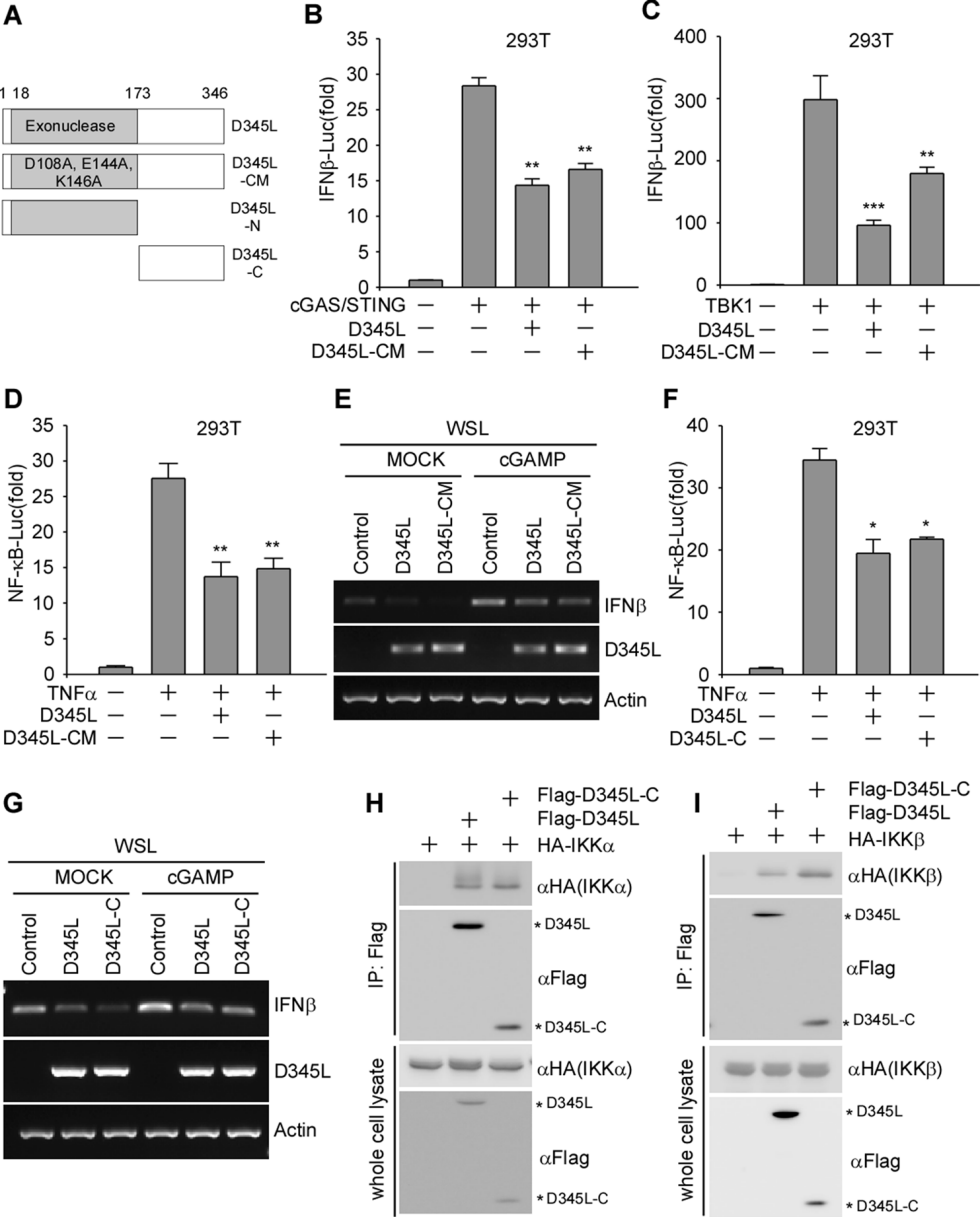
**pD345L-mediated inhibition of NF-κB signalling is independent of its exonuclease activity.** **A** A schematic representation of the D345L catalytic activity mutant (D345L-CM), N-terminal mutant (D345L-N), and N-terminal deletion mutant (D345L-C). **B** 293 T cells transfected with IFNβ-Luc, pCMV-RL, pcDNA3.1-HA-cGAS and pcDNA3-2xFlag-STING, along with p3XFLAG-CMV-D345L, p3XFLAG-CMV-D345L-CM or empty vector for 24 h, were collected to measure the luciferase activity. **C** The experiment was performed as in Panel B except using pcDNA4-HA-TBK1. **D** 293 T cells transfected with NF-κB-Luc and pCMV-RL along with p3XFLAG-CMV-D345L, p3XFLAG-CMV-D345L-CM or empty vector for 24 h followed by treatment with TNFα (40 ng/mL) for 6 h were collected to measure the luciferase activity. **E** WSL cells transfected with p3XFLAG-CMV-D345L or p3XFLAG-CMV-D345L-CM or empty vector for 30 h followed by stimulation with 2′3′-cGAMP (5 μg/mL) for 4 h and harvested for RNA extraction. Semiquantitative RT–PCR assays were carried out to detect IFNβ, D345L and Actin mRNA expression levels. **F** The experiment was performed as for **D**, except that p3XFLAG-CMV-D345L-C was transfected. **G** The experiment was performed as for Panel E, except that p3XFLAG-CMV-D345L-C was transfected. 293 T cells were co-transfected with Flag-D345L, Flag-D345L-C and HA-IKKα (**H**), HA-IKKβ (**I**) or empty vector for 36 h, and the whole-cell lysates were immunoprecipitated with Flag antibody. The immunocomplexes were analysed by immunoblotting with the indicated antibodies.

The pD345L protein contains an N-terminal 5’ → 3’ exonuclease domain and shows strong similarity with lambda phage exonuclease, while its C-terminal function remains unknown [12]. To investigate whether the inhibition of NF-κB activity relies on pD345L exonuclease activity, a D345L catalytic mutant (D345L-CM) was constructed (Figure 6A). In D345L-CM, aspartic acid 108 (D108), glutamic acid 144 (E144), and lysine 146 (K146) were replaced with alanine (A) based on the residues in the lambda phage exonuclease catalytic centre [30]. A dual-luciferase reporter assay was conducted on cells transfected with of D345L or D345L-CM along with cGAS/STING or TBK1 and showed that pD345L-CM was still able to suppress the activation of the IFNβ promoter (Figures 6B and C) and NF-κB-Luc (Figure 6D). Consistent with these findings, stimulation of IFNβ mRNA expression by 2′3’cGAMP was attenuated by both pD345L and pD345L-CM in WSL cells (Figure 6E). To confirm these results, D345L N- or C-terminal truncation mutants with or without the exonuclease domain (D345L-N and D345L-C) were constructed. Unfortunately, D345L-N could not be expressed in 293 T cells. Therefore, D345L-C was used for the NF-κB luciferase reporter assay and RT–PCR. The results showed that pD345L-C was still capable of inhibiting NF-κB signalling (Figure 6F) and IFNβ production (Figure 6G). Finally, a coimmunoprecipitation assay was performed to detect the interaction between pD345L-C and IKK. The results showed that HA-IKKα and HA-IKKβ were detected in anti-Flag antibody-precipitated pD345L or pD345L-C (Figures 6H and I).

Taken together, these results indicated that the ASFV pD345L protein blocks NF-κB signalling by interacting with IKK and subsequently suppressing its kinase activity towards IκBα.

## Discussion

Since the expansion of ASFV-affected areas into Asian countries, especially China, an effective vaccine is urgently needed more than ever. However, the development of an effective vaccine is largely hindered due to the limited knowledge of protective antigens and the interaction between ASFV and host immune responses. NF-κB signalling plays a critical role in the innate immune system by controlling the transcription of antiviral genes, including IFNs, cytokines, chemokines and their modulators and immunoreceptors, and genes involved in the cell cycle, apoptosis and stress response. Therefore, viruses have evolved multiple strategies to disrupt the NF-κB signalling cascade to guarantee efficient replication. The kinase activity of the IKK protein complex (IKKα/β/γ) is essential for NF-κB activation and has thus become a common target of many viruses. For example, the influenza A virus NS1 protein targets the kinase domain (KD) of IKKα/β, blocking IKKβ-mediated phosphorylation and degradation of IκBα in the canonical pathway and IKKα-mediated processing of the precursor protein p100 to p52 in the noncanonical pathway [31]. In addition, the vaccinia virus (VACV) B14 protein inhibits IKKβ activation by binding to its KD and its scaffold and dimerization domain (SDD) [32–34]. Moreover, the molluscum contagiosum virus (MCV) MC160 protein is capable of inhibiting IKK complex formation [35].

In this study, we found that the ASFV-encoded lambda-like exonuclease pD345L is an inhibitor of cGAS/STING-mediated NF-κB signalling. As a result, overexpression of pD345L obviously suppressed cGAMP-induced transcript levels of IFNβ and inflammatory cytokines (IL-1α, IL-6, IL-8, and TNFα) (Figure 2). Importantly, we demonstrated that along the NF-κB signalling cascade, pD345L specifically targets the IKK complex by interacting with the KD and HLH domains of IKKα (Figure 4H) and the LZ domain of IKKβ (Figure 4I). Although the structures of IKKα and IKKβ are similar, the conformations of the IKKα/β truncation mutants may not be identical. Each protein has a distinct binding preference. IKKα and IKKβ share only 52% amino acid similarity, which may cause IKKα/β differential binding to pD345L. Previous reports showed that the LZ domain is required for proper formation of IKKα and IKKβ homodimers or heterodimers, and the LZ domain or HLH motif defective mutants of IKKα and IKKβ block their kinase activities [29, 36, 37]. Interestingly, we found that in the presence of pD345L, IκBα phosphorylation by IKK was decreased (Figures 5A and B). To our knowledge, this is the first time that any ASFV protein was able to block IKKα/β kinase activity through protein–protein interactions.

In addition to NF-κB-dependent targets, IKK also regulates a number of substrates involved in regulating cell growth, apoptosis, autophagy and metabolism [38]. For example, IKKβ promotes the transcription of antiapoptotic genes in the canonical NF-κB pathway; in contrast, IKK inhibits apoptosis by phosphorylating the proapoptotic proteins PUMA at serine 10 and Bad at serine 26 [39, 40]. In addition, active IKK subunits stimulate autophagy, and knockout of IKKβ inhibits the activation of autophagy in mice [41]. The autophagy-related proteins ATG16L1 (autophagy-related 16-like 1) and AMBRA1 (autophagy and beclin-1 regulator 1) were identified as IKK substrates [42, 43]. It has been shown that precise manipulation of host cell apoptosis by ASFV is critical to accomplish different infection stages of its life cycle [44–46]. However, autophagy is not required for ASFV replication, and autophagosome formation is inhibited upon ASFV infection [47]. Therefore, by modulating IKK activity, pD345L possibly regulates multiple biological pathways. Whether pD345L suppresses the kinase activity of IKK towards PUMA, Bad or other substrates to regulate apoptosis or autophagy in ASFV-infected cells needs to be further explored.

To determine the effect of D345L deletion on virus-induced IFNβ responses, the generation of D345L-deficient ASFV was attempted but failed, consistent with a previous report [12], suggesting the essential role of pD345L in ASFV replication. Conditional deletion of D345L will be attempted in further studies [48, 49]. pD345L is a lambda-like exonuclease, and such proteins also found in phycodnaviruses, mimiviruses and bacteriophages are implicated in DNA repair and chromosome recombination [4, 30]. Conventional vaccine development approaches have been shown to be inapplicable to ASFV [50, 51]. Neither subunit nor DNA vaccine provides efficient protection [52]. Gene-deleted live-attenuated vaccine (LAV) candidates render adequate protection by eliciting both humoral and cellular immunity, but the establishment of chronic or persistent infection in vaccinated animals and the risk of recombination with field strains are unsolved key issues. Here, we showed that D345L is not only an essential gene for ASFV replication but also inhibits the host immune response, which may explain why LAVs maintain immune evasion ability but to a lesser extent. To overcome LAV-induced persistent infection, one potential strategy is to generate replication-defective viruses, thus limiting or abrogating viral replication in animals by targeting replication-essential viral genes. Given the dual role of pD345L in viral replication and immune evasion, it may constitute a potential target for the development of replication-defective viral vaccines.

## Data Availability

All data generated or analysed during this study are included in this published article.
